# Transcriptome Analysis of *Campylobacter jejuni* and *Campylobacter coli* during Cold Stress

**DOI:** 10.3390/pathogens12070960

**Published:** 2023-07-21

**Authors:** Anand B. Karki, Bhuwan Khatri, Mohamed K. Fakhr

**Affiliations:** 1 Department of Biological Science, The University of Tulsa, Tulsa, OK 74104, USA; 2 Genes and Human Disease Research Program, Oklahoma Medical Research Foundation, Oklahoma City, OK 73104, USA

**Keywords:** *Campylobacter*, cold stress, transcriptome, retail meat, poultry

## Abstract

*Campylobacter* spp. are known to cause campylobacteriosis, a bacterial disease that remains a public health threat. *Campylobacter* spp. are prevalent in retail meat and liver products, and the prolonged survival of *Campylobacter* in the low temperatures needed for storage is a challenge for food safety. In this study, RNA-seq was used for the analysis of the *C. coli* HC2-48 (Cc48) and *C. jejuni* OD2-67 (Cj67) transcriptomes at 4 °C in a nutrient-rich medium (chicken juice, CJ) and Mueller–Hinton broth (MHB) for 0 h, 0.5 h, 24 h and 48 h. Differentially expressed genes (DEGs) involved in flagellar assembly were highly impacted by low temperatures (4 °C) in *C. coli* HC2-48, whereas genes related to the ribosome and ribonucleoprotein complex were modulated for *C. jejuni* OD2-67 at 4 °C. Most of the DEGs in cells grown at 4 °C in the two medium formulations were not significantly expressed at different incubation times. Although more DEGs were observed in CJ as compared to MHB in both *Campylobacter* strains, the absence of common genes expressed at all incubation times indicates that the food matrix environment is not the sole determinant of differential expression in *Campylobacter* spp. at low temperatures.

## 1. Introduction

*Campylobacter* spp. are microaerobic, thermophilic bacteria (optimal temperature ~42 °C) that lack cold-shock-response genes; despite this limitation, *Campylobacter* strains survive at the low temperatures used for food storage conditions [1]. The microenvironment has a profound influence on the survival of *Campylobacter* [2]; for example, a nutrient-rich environment containing meat or liver juice facilitates the survival of *Campylobacter* strains at low temperatures and may contribute to the high frequency of *Campylobacter* spp. in retail meats and liver products during slaughter, processing and storage [1,3]. We previously reported the high prevalence of *C. jejuni* and *C. coli* strains in retail liver, chicken, pig and beef products [4,5,6]. Aerotolerance and co-contaminants such as *Staphylococcus aureus* also improve *Campylobacter* survival in adverse environments including aerobic conditions and low temperatures [7,8]. 

A prior study reported that *Campylobacter* survival at low temperatures is presumably an active process where changes occur in lipids, oligosaccharides and polysaccharides [9]. In contrast, another report suggested that adaptation to low temperatures is a passive mechanism where various genes (e.g., *clpB, trxC, perR*) and two-component regulatory systems (RacRS) were essential for survival in a nutrient-rich or minimal medium [10]. Other genes that might contribute to *Campylobacter* survival at low temperatures include *sodB*, *luxS* and genes related to motility, chemotaxis, energy production/conversion, amino acid transport/metabolism and lipid transport/metabolism [9,11,12,13]. Furthermore, the acquisition of cryoprotectant molecules might also contribute to survival at low temperatures [10,13]. 

It is well-established that microorganisms cope with environmental adversity by altering gene expression. Global changes in *Campylobacter* gene expression were influenced by medium, days of incubation, temperature and atmospheric conditions [9,12]. Several microarray studies documented changes in the *C. jejuni* transcriptome at low temperatures [9,11,12,13]; however, only one report used RNA-seq [11], which is a more sensitive and accurate approach than the microarray analysis for genomic expression studies [14]. The global response of *C. coli* strains to low temperatures remains unclear, although a prior report suggested that *Campylobacter* species respond differentially to environmental stress [15]. 

In the current study, RNA-seq was used to explore gene expression of *C. jejuni* and *C. coli* during cold stress. The transcriptomes of *C. coli* and *C. jejuni* strains were analyzed at 4 °C in chicken juice (CJ) and Mueller Hinton Broth (MHB) at 0 h, 0.5 h, 24 h and 48 h of incubation and compared to controls that were incubated in MHB at 42 °C. Food matrix environments and incubation times affected the transcriptomes of *Campylobacter* strains during cold stress. Meanwhile, transcriptomic analysis indicated that cold shock responses might differ between *Campylobacter* spfecies. 

## 2. Methodology

### 2.1. Preparation of Retail Meat and Liver Juices 

Retail chicken was used as a food model, and MHB was used as a medium control. CJ was prepared as described previously [1]. Briefly, frozen, whole chickens were purchased from retail stores, thawed overnight at room temperature, and juices were isolated as described in [1]. The absence of contaminants in CJ was confirmed by subculturing in Mueller Hinton agar (MHA) supplemented with 5% laked horse blood [1]. Sterile CJ was stored at −20 °C until needed for experiments. Frozen CJ was thawed at 4 °C overnight, and 7 mL aliquots were dispensed up to the rim of polystyrene culture tubes (5 mL); CJ was then stored at 4 °C until it was used for survival assays. Freshly prepared MHB was used as a control and maintained at 4 °C in culture tubes. 

### 2.2. Bacterial Strains and Growth Conditions

Two *Campylobacter* strains, *C. jejuni* OD2-67 (Cj67) and *C. coli* HC2-48 (Cc48), were used in this study; these two strains were previously isolated, characterized and sequenced in our laboratory [4,6,16,17]. *Campylobacter* strains were cultured from frozen stocks (−70 °C) in MHA containing 5% laked horse blood and supplemented with antibiotics (Bolton selective supplement, Himedia). Strains were grown at 42 °C in microaerobic conditions (CampyGen^TM^ 3.5L, Thermo Scientific, Waltham, MA, USA) for 48 h, subcultured into freshly prepared MHB (75 mL) for 16 h at 42 °C in microaerobic conditions and harvested during log phase as described below. 

### 2.3. Preparation of Cells for RNA Isolation 

Bacterial cells (log phase cultures) were pelleted at 6000 rpm for 10 min and suspended in freshly prepared MHB to an OD_600_~0.5. Prepared bacterial suspensions were maintained at 42 °C in microaerobic conditions for 2 h. For control samples at 42 °C, Cc48_42 (*C. coli* HC2-48) and Cj67_42 (*C. jejuni* OD2-67), cells (150 µL bacterial suspensions) were collected from cultures incubated at 42 °C in microaerobic condition. Control samples were then immediately mixed with TRI reagent (Zymo Research, Irvine, CA, USA) (700 µL) and maintained at −70 °C until RNA was isolated. 

For experiments documenting survival at 4 °C, bacterial cell suspensions (0.7 mL) maintained at 42 °C in microaerobic condition for 2 h were added to 7 mL pre-chilled (4 °C) MHB or CJ and mixed. Inoculated medium (350 µL) was then removed and mixed with three volumes TRI reagent and designated as the 0 h sample for *C. coli* HC2-48 (Cc48_MHB_0h and Cc48_CJ_0h) and *C. jejuni* OD2-67 (Cj67_MHB_0h and Cj67_CJ_0h). Sample names were assigned to include strain name (Cc48 or Cj67)_medium (MHB or CJ)_time of incubation at 4 °C (0 h, 0.5 h, 24 h or 48 h). Remaining inoculated media were then incubated at 4 °C. A similar approach was used to collect samples at 0.5 h (Cc48_MHB_0.5h, Cc48_CJ_0.5h, Cj67_MHB_0.5h and Cj67_CJ_0.5h), 24 h (Cc48_MHB_24h, Cc48_CJ_24h, Cj67_MHB_24h and Cj67_CJ_24h) and 48 h (Cc48_MHB_48h, Cc48_CJ_48h, Cj67_MHB_48h and Cj67_CJ_48h) (Table S1). Collected samples were mixed with TRI reagent and stored at −70 °C until needed for RNA isolation. All experiments were carried out in triplicate.

### 2.4. RNA Extraction 

Frozen samples were allowed to reach room temperature, and cells were disrupted with vigorous vortexing. Total RNA was extracted with the Directzol RNA isolation kit (Zymo Research, Irvine, CA, USA) and TRI reagent as recommended by the manufacturer. On-column DNA digestion was performed using instructions provided with the Directzol RNA isolation kit followed by an additional two-step DNase treatment (TURBO DNA-free Kit, Invitrogen, Vilnius, Lithuania) to eliminate any contaminating DNA. Total RNA was quantified with the Nanodrop spectrophotometer and the Qubit™ RNA HS Assay Kit (Invitrogen, Eugene, OR, USA). Absence of genomic DNA contamination in RNA samples was confirmed by PCR with primers of housekeeping genes *glyA* and *aspA* as described previously [7]. The quality of RNA was checked by denaturing RNA electrophoresis in agarose gels [18]. RNA samples (5–10 µg) were treated with the Ribominus Transcriptome Isolation Kit (bacteria) (Invitrogen, Carlsbad, CA, USA) for removal of ribosomal RNA and re-analyzed by denaturing RNA electrophoresis. Prior to preparation of cDNA libraries, triplicate samples from replicated biological experiments were mixed together in equal concentration. 

### 2.5. cDNA Library Preparation, Sequencing and Expression Analysis

Approximately 100 ng of purified mRNA samples were used as starting material for cDNA libraries using the Illumina TrueSeq Stranded mRNA Library Prep kit as recommended (Illumina, San Diego, CA, USA) with minor modifications. The procedure for selective enrichment of mRNA was skipped, and cDNA libraries were quantified with the Qubit dsDNA HS Assay kit. The quality of cDNA libraries was determined using the Agilent High Sensitivity DNA kit and Agilent Bioanalyzer 2100 (Agilent, Santa Clara, CA, USA). Prepared cDNA libraries were sent to service provider (Quick Biology Inc., Monrovia, CA, USA) for sequencing in the Illumina HiSeq 4000 platform with a read length of 2 × 150 bp (paired-end run). All the original sequence reads for this experiment were deposited in GenBank and are accessible in the Sequence Read Archive (SRA) database within Bio project id: PRJNA828109.

RNA sequences were analyzed with the CLC Genomics Workbench v. 20 as described previously [19]. Raw sequence reads were trimmed to remove adapter sequences and ambiguous reads as needed, and all reads <10 bp were discarded. Genomic sequences of *C. jejuni* OD2-67 (NZ_CP014744, NZ_CP014745 and NZ_CP014746) and *C. coli* HC2-48 (NZ_CP013034 and NZ_CP013035) were downloaded from Ref Seq (https://www.ncbi.nlm.nih.gov/refseq, accessed on 9 March 2020) and used as references. Sequence reads that mapped to rRNAs from bacteria and chicken were removed from downstream analyses. Read counts were normalized and differential expression was conducted in the CLC Genomic Workbench v. 20 with default settings. All noncoding sequences, pseudogenes, and frameshifted genes were excluded from analysis. Differentially expressed genes (DEGs) with fold-change values ≥ 1.5 or ≤−1.5 and false discovery rates (FDR) < 0.05 were considered significant. Heatmaps were created using the ComplexHeatmap package (Bioconductor), and Venn diagrams were created with Venny 2.1 (https://bioinfogp.cnb.csic.es/tools/venny, accessed on 9 March 2020). Genome-wide functional annotation of *C. jejuni* OD2-67 and *C. coli* HC2-48 was carried out with the noncurated eggNOG v. 5.0 database [20] (http://eggnog-mapper.embl.de, accessed on 9 March 2020) with e-value of 0.001, seed ortholog score of 80 and both query and subject cover cutoff value of 60%. Gene annotation and gene enrichment analysis for common DEGs were conducted using STRING v. 11 (https://string-db.org, accessed on 9 March 2020) with default settings. Schematic representations of expression levels in different functional groups were executed with the Circos Table viewer (http://mkweb.bcgsc.ca/tableviewer, accessed on 9 March 2020). 

Nucleotide sequence similarity analyses (BlastN, BLAST Atlas) were conducted with genomes of *C. coli* HC2-48 and *C. jejuni* OD2-67 using files retrieved from RefSeq in the Gview server (https://server.gview.ca, accessed on 9 March 2020); the settings included e-values of 1e-10, alignment length cutoff value of 100 bp and identity cutoff values of 80%. Other genes with similar names and functions were included as orthologous genes. 

### 2.6. Validation of Differential Gene Expression by qRT-PCR

RNA samples from biological experiments (triplicates) were extracted as described above for RNA isolation. The primers used for target genes and endogenous controls (*ilvC* and *slyD*) are listed in Table S2. Quantitative real-time PCR (qRT-PCR) was conducted using the QuantiTect SYBR Green RT-PCR kit (Qiagen) in MicroAmp Fast 96-well reaction plates (Applied Biosystems) as described in [7]. One-step qRT-PCR cycles were executed using the StepOne Real-Time PCR System (Applied Biosystems). Relative quantification of gene expression was conducted using the 2^−∆∆Ct^ method [21], and statistical analysis was performed using GraphPad Prism v. 9 (https://www.graphpad.com/scientific-software/prism, accessed on 9 March 2020). 

## 3. Results

### 3.1. Overview of RNA-Seq

Raw sequence reads were trimmed for adapters using the CLC Genomic Workbench v. 20 with default settings; all ambiguous reads and reads less than 10 bp were discarded. Among the trimmed sequence reads from microaerobic incubation of *C. coli* HC2-48 (sample Cc48_42) and *C. jejuni* OD2-67 (Cj67_42) at 42 °C, approximately 97% mapped to reference sequences. Similarly, over 93% of sequence reads from MHB samples of *C. coli* HC2-48 (Cc48_MHB_0h, Cc48_MHB_0.5h, Cc48_MHB_24h and Cc48_MHB_48h) and *C. jejuni* OD2-67 (Cj67_MHB_0h, Cj67_MHB_0.5h, Cj67_MHB_24h and Cj67_MHB_48h) mapped to reference sequences. However, only 17% to 39.14% of sequence reads from samples incubated in CJ (*C. coli* samples Cc48_CJ_0h, Cc48_CJ_0.5h, Cc48_CJ_24h and Cc48_CJ_48h; *C. jejuni* samples Cj67_CJ_0h, Cj67_CJ_0.5h, Cj67_CJ_24h and Cj67_CJ_48h) were mapped to reference sequences. Details regarding mapping percentages of individual samples are listed in Table S1. 

### 3.2. Influence of Temperature on Gene Expression (4 °C vs. 42 °C)

**Figure 1:**
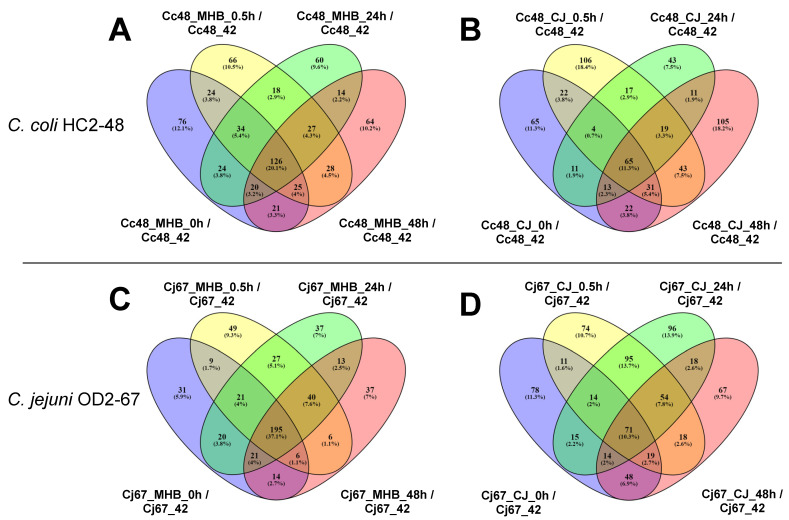
*Campylobacter* genes with significantly different expression values (fold-change ≥ 1.5 or ≤−1.5 fold and FDR < 0.05) at 4 °C vs. 42 °C. *C. coli* HC2-48 (**A**,**B**) and *C. jejuni* OD2-67 (**C**,**D**) were incubated in MHB (**A**,**C**) and CJ (**B**,**D**). {Sample name for samples incubated at 4 °C: strain(Cc48 or Cj67)_medium (MHB or CJ)_incubation time at 4 °C (0 h or 0.5 h or 24 h or 48 h), sample name for control sample incubated at 42 °C: strain(Cc48 or Cj67)_incubation temperature (42)}.

From a total of 1651 analyzed genes from *C. coli* HC2-48, 831 were differentially expressed at 4 °C vs. 42 °C (fold-change ≥ 1.5 or ≤−1.5 fold; FDR < 0.05) in MHB and/or CJ at one or more sampling times (0 h, 0.5 h (30 min), 24 h, 48 h). Similarly, 808 genes out of 1774 were differentially expressed at 4 °C vs. 42 °C in *C. jejuni* OD2-67 in MHB and/or CJ at one or more different sampling times. Details regarding the number of genes upregulated or downregulated at the four incubation times in MHB and CJ are shown in Table 1 and Figure 1.

*C. coli* HC2-48: Only 49 genes in HC2-48 showed significant levels of differential expression in both MHB and CJ at all incubation times (Table 2 and Table S3). The 13 upregulated DEGs included *ppa* (inorganic diphosphatase), *petC* (cytochrome C1), *rplM, rplQ, rpsI* (ribosomal proteins) and the lipolysaccharide (LPS) gene, *lptF* (LptF/LptG family permease). The 36 downregulated genes included ten associated with flagella assembly (*flhB, fliD, flgL, flgG, flaG, fliS, flgH, flgB, flgF, flgK, flgM, flgN*). Meanwhile, one hundred twenty-six genes were differentially expressed in MHB at 4 °C vs. 42 °C in all incubation times (Table S4), whereas sixty-five genes were differentially expressed in CJ at 4 °C vs. 42 °C in all four incubation times (Table S5). 

Six genes were significantly upregulated in MHB but not in CJ at all incubation times. These DEGs included the following: *lptD*, LPS assembly protein; *tolA*, TonB C-terminal domain-containing protein; *murE*, UDP-N-acetylmuramoyl-L-alanyl-D-glutamate-2, 6-diaminopimelate ligase; *AR446_RS05150*, hypothetical protein; *AR446_RS04310*, DUF342- domain-containing protein; and *lptA*, LPS periplasmic transport protein. Two genes, *rpmL* (50S ribosomal protein L35) and *shlA* (filamentous hemagglutinin N-terminal-domain-containing protein) were downregulated in MHB but not in CJ. However, no genes were identified that were significantly expressed in CJ but not in MHB. 

*C. jejuni* OD2-67: Fifty eight genes showed significant differential expression in MHB and CJ at 4 °C vs. 42 °C in all incubation times (Table 3 and Table S6). In MHB, 195 genes were differentially expressed at 4 °C vs. 42 °C in all incubation times (Tables S7 and S8), and 71 genes were significantly expressed in all CJ samples (Table S9). Genes that were expressed in MHB but not in CJ included four upregulated genes (*pgpA, cmeR, tssJ and macB1*) and four downregulated genes (*ndk, corC*, *A0W68_RS03455* (HAD-IB family hydrolase) and *A0W68_RS06390* (hypothetical protein)). Two genes were significantly expressed in CJ but not in MHB; these included upregulated *corA* (magnesium/cobalt transporter) and downregulated *A0W68_RS09110* (NYN-domain-containing protein). 

### 3.3. Functional Group Assignments of DEGs 

**Figure 2:**
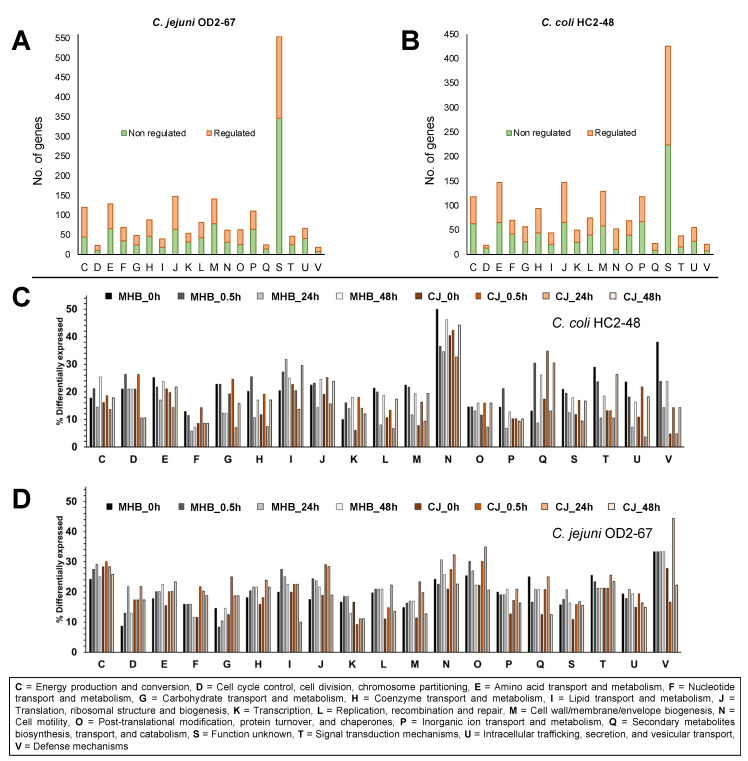
Schematic representation of significantly regulated and nonregulated genes (for 4 °C vs. 42 °C) at one or more incubation times or medium formulations. Numbers of genes are shown for *C. coli* HC2-48 (**A**) and *C. jejuni* OD2-67 (**B**). The percentage of DEGs in each functional group is shown for sampling time and media for *C. coli* HC2-48 (**C**) and *C. jejuni* OD2-67 (**D**). Sample names represent medium (MHB or CJ) and sampling time (0 h, 0.5 h, 24 h or 48 h) for each *Campylobacter* strain. COG functional groups are described below panel (**D**).

Among DEGs expressed (4 °C vs. 42 °C) at one or more incubation times, 10.83% of *C. coli* HC2-48 and 25.62% of *C. jejuni* OD2-67 genes were assigned to the ‘unknown function’ category (Figure 2A,B, group S).

The highest percentage of differential expression in *C. coli* HC2-48 was observed for cell motility (group N), and this group assignment was consistent for all incubation times and medium formulations (Figure 2C). For *C. jejuni* OD2-67, genes from energy production/conversion, cell motility, post-translational modification/chaperones and defense mechanisms (groups C, N, O and V, respectively) were differentially expressed at 4 °C in both media (Figure 2D). 

**Figure 3:**
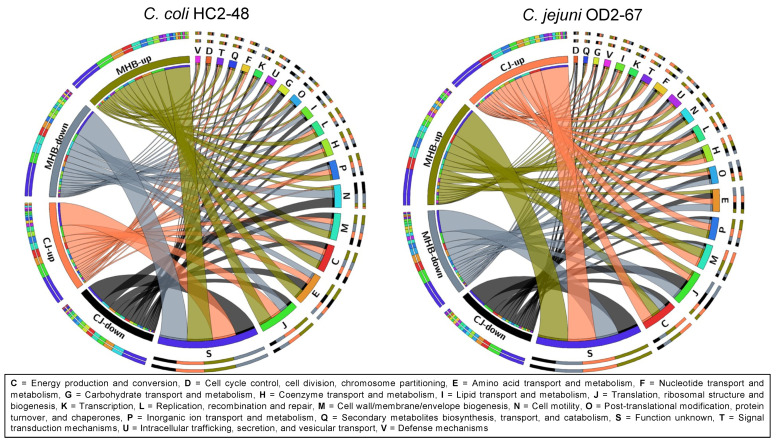
Differential expression of genes (for 4 °C vs. 42 °C) in 19 COG functional groups. Log_2_ fold-change values are shown for genes with significant expression at one or more incubation times or media.

**Figure 4:**
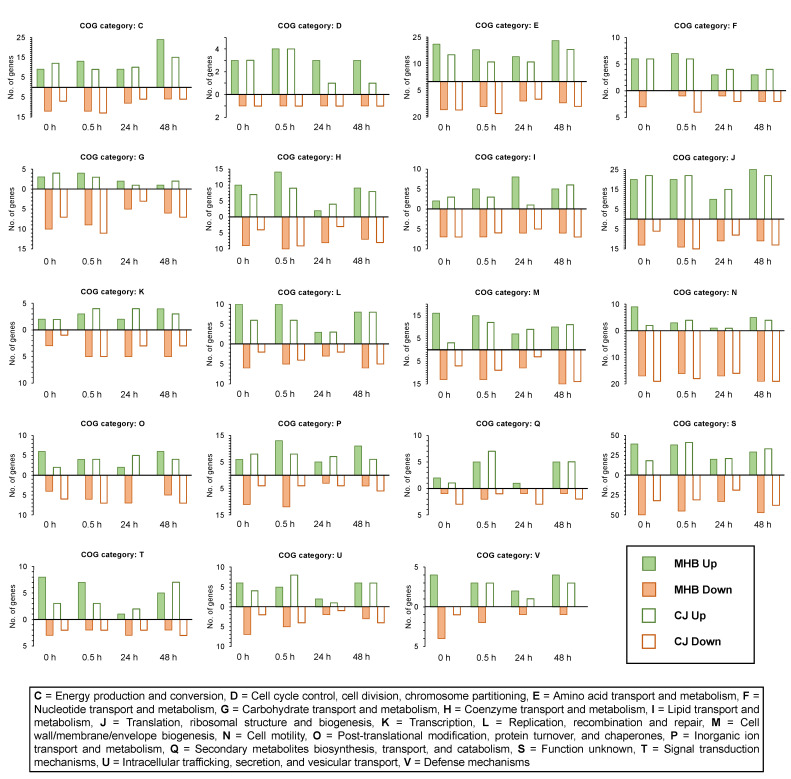
Numbers of significantly upregulated and downregulated DEGs in *C. coli* HC2-48 incubated at 4 °C and four different incubation times in MHB and CJ as compared to expression at 42 °C in MHB. The COG functional group assignments are listed below the figure.

**Figure 5:**
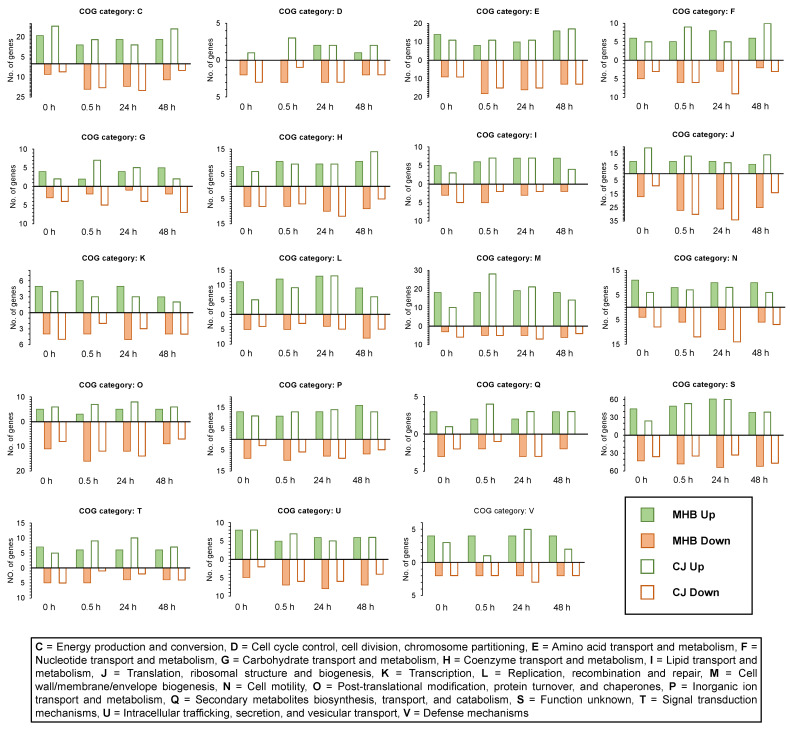
Number of significantly upregulated or downregulated DEGs in *C. jejuni* OD2-67 at 4 °C for four different times in MHB and CJ and compared to expression at 42 °C in MHB. The COG functional group assignments are listed below the figure.

In general, larger fold-change values were observed for upregulated *C. coli* HC2-48 genes in both media than for downregulated genes; however, exceptions were genes in the following functional groups: N (cell motility), I (lipid transport/metabolism), O (post-translational modification/chaperones) and G (carbohydrate transport/metabolism) (Figure 3 and Figure 4). Accumulative expression values (fold-change) for the genes related to amino acid transport/metabolism (group E) in *C. coli* were greater for upregulated genes than downregulated genes in MHB, but it was found to be the opposite in CJ. In *C. jejuni* OD2-67, downregulated genes in functional group J (translation/ribosome structure, O (post-translational modification, chaperones) and D (cell cycle control, cell division, chromosome partitioning) had larger fold-changes values than upregulated genes (Figure 3 and Figure 5). However, accumulative fold-change values of upregulated genes in functional groups C, M, P, H, L, U, F, T, I, V and Q for both media were higher than downregulated genes from respective functional groups (Figure 3 and Figure 5). 

Among the DEGs of *C. coli* HC2-48 found in both media (CJ and MHB) and at all incubation times, gene enrichment analysis in STRING found significant enrichment (FDR < 0.05) in the KEGG pathway for flagellar assembly (https://www.genome.jp/dbget-bin/www_bget?ko02040, accessed on 9 March 2020). Although *C. jejuni* OD2-67 lacked a KEGG pathway that was enriched for all incubation times and media, Gene Ontology analysis showed that the ribosome/ribonucleoprotein complex was significantly enriched (FDR < 0.05) for all sampling times in MHB. 

### 3.4. Validation of RNA-Seq Data by qRT-PCR

**Figure 6:**
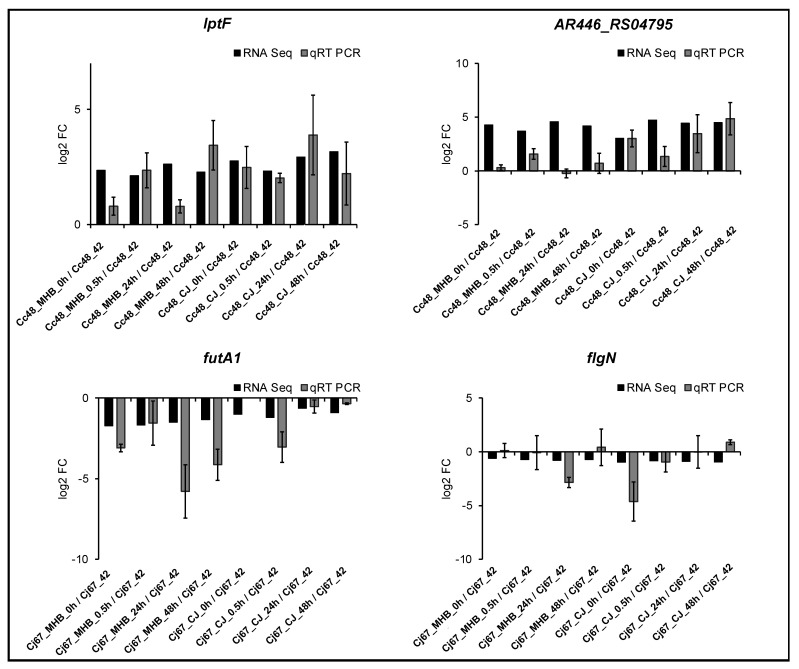
qRT-PCR validation of RNA-seq results. Two genes that were upregulated in RNA-seq data, *lptF* and *AR446_RS04795*, were used to follow expression in *C. coli* HC2-48, and two downregulated genes, *futA1* and *flgN*, were used to validate RNA-seq data for *C. jejuni* OD2-67. Samples were incubated in MHB or CJ medium at different incubation times (0 h, 0.5 h, 24 h and 48 h), and expression was compared to control strains cultivated in MHB at 42 °C with microaeration. Triplicate biological replicates were used for qRT-PCR analysis, and error bars represent the standard error of means (mean ± SEM).

Four DEGs with distinct expression patterns were used to confirm RNA-seq data by qRT-PCR. Two upregulated genes, namely *lptF* and *AR446_RS04795*, were chosen to validate RNA-seq results for *C. coli* HC2-48, and the downregulated genes *futA1* and *flgN* were used to validate results for *C. jejuni* OD2-67. The expression profiles obtained by qRT-PCR were consistent with results obtained with RNA-seq, indicating that the RNA-seq data are reliable (Figure 6).

### 3.5. Common DEGs in C. coli and C. jejuni (4 °C vs. 42 °C)

Analysis of RNA-seq data indicated that 1386 and 1385 genes from *C. coli* HC2-48 and *C. jejuni* OD2-67, respectively, were shared orthologs. However, only 11 genes had fold-change values with FDR < 0.05 at all incubation times and in both media and strains (Table 4 and Table S10), but only two genes, *fliS* and *flgN*, were differentially expressed with significant fold change (fold-change values ≥ 1.5 or ≤−1.5 and FDR < 0.05 for both strains at all data points in both media.

Common DEGs with significance differences (fold-change ≤−1.5 fold) in CJ included downregulated *hemE*, *flhB*, *flaG*, *fliS* and *flgN* at all incubation time points. Three genes including *mug* (DNA-deoxyinosine glycosylase), *amaA* (AI-2E family transporter) and *AR446_RS04310/A0W68_RS04275* (DUF342-domain-containing protein) were upregulated in MHB for both strains at all times. Common downregulated genes for the two strains in MHB included *cysK* (cysteine synthase A), *rpmI, cfa* (class I SAM-dependent methyltransferase) and the flagellar genes *fliS*, *flgG* and *flgN*. 

Forty-one genes exhibited a positive fold change in expression (significant or nonsignificant) in *C. coli* HC2-48 but had negative fold-changes values (significant or nonsignificant) in *C. jejuni* OD2-67. This group included genes involved in amino acid transport and metabolism (e.g., *ilvF, ilvK, hisD, hisF*, *cysE*, *pfs, dapE*) and translation and ribosomal structure (*rimP, thrS, rpiL, mnmE, leuS, pnp, tyrS, tsaD, rpsD, rpsC, rplW*). Fifty-eight genes exhibited positive fold-change values for *C. jejuni* OD2-67 but negative fold-change values for *C. coli* HC2-48. This group included genes for amino acid transport and metabolism (*aroQ, aroB, proA, proC, sstT*) and genes involved in cell wall and membrane biogenesis (*lpxB, kpsC, murC, rfaD, galU, murB*). 

### 3.6. Influence of Medium Formulation on Gene Expression (CJ vs. MHB)

**Figure 7:**
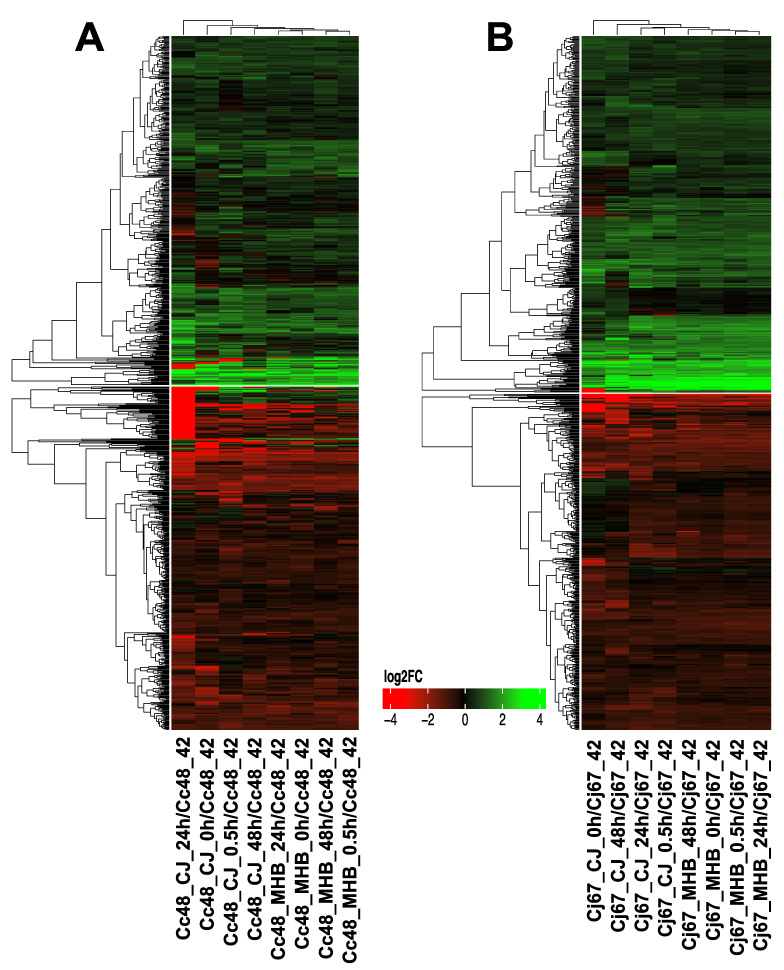
Heatmap showing DEGs with significant fold-change values at one or more sampling times or medium formulations of (**A**) *C. coli* HC2-48 and (**B**) *C. jejuni* OD2-67. Sample designations are provided below the heatmaps. Each sample was compared to expression levels in the control (MHB at 42 °C, with microaerobic conditions).

**Figure 8:**
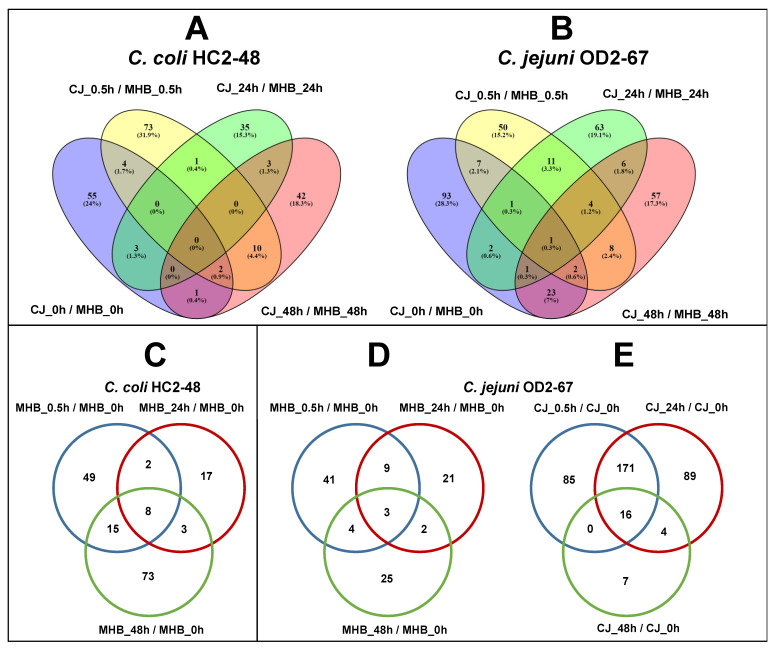
Differentially expressed genes in CJ with significant fold-change values (≥1.5 or ≤−1.5, FDR < 0.05) at different incubation times at 4 °C as compared to expression in MHB (**A**,**B**). Venn diagrams (**C**,**D**) showing DEGs with significant expression at different incubation times in MHB as compared to expression in MHB at 0 h. DEGs at different incubation times in CJ as compared to expression in CJ at 0 h (**E**). Panels: (**A**,**C**) *C. coli* HC2-48, (**B**,**D**,**E**) *C. jejuni* OD2-67. Sample names for each strain include medium (MHB or CJ)_incubation time at 4 °C (0 h or 0.5 h or 24 h or 48 h).

Although most genes in the two *Campylobacter* spp. had similar expression patterns (Figure 7), some genes were differentially expressed in the different medium formulations (CJ vs. MHB) (Figure 8). In *C. coli* HC2-48, 229 DEGs were significantly expressed in CJ vs. MHB at one or more sampling times (Cc48_CJ_0h/Cc48_MHB_0h, Cc48_CJ_0.5h/Cc48_MHB_0.5h, Cc48_CJ_24h/Cc48_MHB_24h and Cc48_CJ_48h/Cc48_MHB_48h). Among these DEGs, no gene was significantly expressed for all sampling times, but 31 genes had positive fold-change values in CJ vs. MHB (Tables S11 and S12), and 23 genes had negative fold-change values in CJ vs. MHB at all sampling times (Table S13). Among the orthologs, *torD* (molecular chaperone TorD family protein) was upregulated (at one or more sampling times) for both *C. jejuni* and *C. coli* in CJ when compared to MHB at 4 °C. Similarly, *katA* (catalase) was downregulated (at one or more sampling times) for both species in CJ as compared to MHB at 4 °C. 

In *C. jejuni* OD2-67, 329 DEGs showed significant expression values in CJ at one or more incubation times when compared to MHB at 4 °C (Cj67_CJ_0/h/Cj67_MHB_0h, Cj67_CJ_0.5h/Cj67_MHB_0.5h, Cj67_CJ_24h/Cj67_MHB_24h and Cj67_CJ_48h/Cj67_MHB_48h). Only one gene *A0W68_RS09275* (EexN family lipoprotein) had significant expression levels (downregulated) at all incubation times (Table S14). Excluding common genes, 45 DEGs had positive fold-change values (significant or nonsignificant) for CJ vs. MHB at all incubation times (Table S15); similarly, 55 DEGs had negative fold-change values (significant or nonsignificant) for CJ vs. MHB at all incubation times (Table S16). 

### 3.7. Influence of Incubation Time on Gene Expression (0.5 h vs. 0 h, 24 h vs. 0 h and 48 h vs. 0 h)

MHB medium and time of incubation: For *C. coli* HC2-48, genes were differentially expressed in MHB at 0.5 h (Cc48_MHB_0.5h), 24 h (Cc48_MHB_24h) and 48 h (Cc48_MHB_48h) at 4 °C compared to 0 h (Cc48_MHB_0h). Seventy-four DEGs (fifty-one upregulated, twenty-three downregulated), thirty (eighteen upregulated, twelve downregulated) and ninety-nine (forty-nine upregulated, fifty downregulated) were identified for Cc48_MHB_0.5h/Cc48_MHB_0h, Cc48_MHB_24h/Cc48_MHB_0h and Cc48_MHB_48h/Cc48_MHB_0h, respectively. Only eight genes were differentially regulated at three incubation times (0.5 h, 24 h and 48 h) compared to 0 h at 4 °C in MHB (Tables S17 and S18). Downregulated genes at 0.5 h included *dnaJ*, *purH, cfrA*, ribosomal genes (*rplS, rpmH, rpmJ*), flagellar genes (*flgJ, flaG*) and *hspR* (heat shock protein). Upregulated DEGs included genes involved in coenzyme transport/metabolism (*mqnA, cca, hemA, ispA, coaD, mob*), translation/ribosomal structure/biogenesis (*prmA, def, fmt*), transport (*AR446_RS06920, AR446_RS07750*, *mdtL, mdtJ*, *AR446_RS04345*, *AR446_RS08130*) and multidrug ABC transporter permease (*AR446_RS00345*, *AR446_RS02400*). At 24 h and 4 °C, *maf*, *metE, ttcA, rub, virB11, nuoE* and *hyaE* were upregulated; downregulated genes included *radA* and *clpX*. Various genes related energy production and conversion (*nuoJ, nuoN, hydD, lldP, napH, dsbD, nuoL*), nucleotide metabolism (*purB, pyrB, pyrF*), ribosomal structure (*rpsC, rpsE, rpsS, prmA*) and transport (*gltJ, tcyB, tctB, dcuA, matE*) were upregulated at 48 h and 4 °C. However, many genes were downregulated at 48 h and 4 °C as compared to 0 h, including those involved in amino acid transport/metabolism (*gmhB, pepF, argC, proB*), ribosomal structure (*gatC, yqeV, rplS, rpmH, rpmJ, rpsP, rpsT*) and heat shock (*hspR*). 

For *C. jejuni* OD2-67 incubated in MHB at 4 °C, 57 genes were differentially expressed at 0.5 h (40 upregulated, 17 downregulated), 35 at 24 h (26 upregulated, 9 downregulated) and 34 at 48 h (12 upregulated, 22 downregulated) as compared to 0 h. Meanwhile, only three DEGs (two upregulated, one downregulated) showed significant fold-change values at 0.5 h, 24 h and 48 h as compared to 0 h in MHB (Figure 8, Table S19); these were upregulated genes *A0W68_RS01005* (hypothetical protein) and *A0W68_RS09275* (EexN family lipoprotein) and the downregulated gene *A0W68_RS02685* (hypothetical protein). At 0.5 h, genes encoding hypothetical proteins, translation-related proteins (*ffmJ, yabO* and *rpmJ*), and inorganic ion transporters (*A0W68_RS00285, A0W68_RS07265* and *modB*) were upregulated. Genes encoding threonine synthase (*thrC*), lipid transport and metabolism (*plsC, pssA*), and ribosomal protein RplT were downregulated at 0.5 h. *A0W68_RS04835* (putative membrane protein), *A0W68_RS06415* (exopolyphosphatase), *lpxH* (UDP-2,3-diacylglucosamine diphosphatase), *hypA* and *vgrG* (type VI secretion system) were among the upregulated genes at 24 h as compared to 0 h. Downregulated genes at 24 h included *ldH* (L-lactate dehydrogenase), *rplT* and *A0W68*_RS01575 (TonB-dependent receptor). At 48 h, *leuA* and *metF* (amino acid transport/metabolism, *tonB* (energy transducer TonB) and *moaC* and *ropZ* were upregulated. Genes that were downregulated at 48 h in MHB included *ldH, virB11, torD, tssE* and many genes encoding hypothetical proteins.

CJ medium and time of incubation: In *C. coli* HC2-48, 61 DEGs (21 upregulated, 40 downregulated) were identified at 0.5 h (Cc48_CJ_0.5h/Cc48_CJ_0h), one upregulated gene was identified at 24 h (Cc48_CJ_24h/Cc48_CJ_0h), and no genes showed significant upregulation at 48 h (Cc48_CJ_48h/Cc48_CJ_0h) (Table S20). Gene *fliK* was the only upregulated gene at both 0.5 hand 24 h. Upregulated genes at 0.5 included *sdaC*, *glnH, pgi, acpP, efp, rim, cmeD, djlA, radA, uraH and virB4*. Downregulated DEGs at 0.5 included genes related to energy production and conversion (*aldA, fdhA, hydA, oorA, mdh, frdA, lutB*), amino acid transport/metabolism (*leuA*), nucleotide transport/metabolism (*atpD, guaB, sucC*), carbohydrate metabolism (*uxaA, glmM, eno, fbaA*) and translation (*rluD, proS, fmt, rplO, rplC, rplD, rplP, rplV, rpsD, rpsJ, rpsK*). 

For *C. jejuni* OD2-67, differential expression of genes in CJ was observed at 0.5 h (Cj67_CJ_0.5h), 24 h (Cj67_CJ_24h) and 48 h (Cj67_CJ_48h) as compared to 0 h (Cj67_CJ_0h). Two hundred seventy-two DEGs (one hundred fifteen upregulated, one hundred fifty-seven downregulated) were identified at 0.5 h, two hundred eighty DEGs (one hundred fourteen upregulated and one hundred sixty-six downregulated) at 24 h and twenty-seven DEGs (eleven upregulated, sixteen downregulated) at 48 h (Table S21). Excluding common genes in Table S22, 61 more genes were upregulated (Table S23) and 110 genes were downregulated (Table S24) at 0.5 hand 24 h. 

## 4. Discussion

*Campylobacter* strains (*C. coli* HC2-48 and *C. jejuni* OD2-67) were isolated from retail liver products {*C. coli* HC2-48 (retail beef liver) and *C. jejuni* OD2-67 (retail chicken liver)} [4,6]. Meanwhile, *C. coli* HC2-48 had been identified as an aerotolerant strain (could survive up to 12 h of aerobic incubation), but *C. jejuni* OD2-67 was found to be aero-sensitive [7]. Both strains harbored chromosome of about 1.7 Mb size, but *C. coli* HC2-48 carried one plasmid (pCCDM1) [17] and *C. jejuni* OD2-67 possessed two plasmids pCJDM67L and pCJDM67S [16]. The megaplasmid pCJDM67L (117 Kb) found in *C. jejuni* OD2-67 harbored genes encoding tetracycline resistance and core genes of type VI secretion system [16]. 

Prolonged survival of *Campylobacter* species at lower temperatures is influenced by multiple factors including nutrient availability, environment and inherent characteristics of the strain [1,2,22,23]. A nutrient-rich environment such as CJ provides a nutritional and protective environment that enhances *Campylobacter* survival at lower temperatures [1]. In a previous study in our laboratory, both *Campylobacter* strains (*C. coli* HC2-48 and *C. jejuni* OD2-67) used in this study could not produce colonies after two days of incubation in MHB medium at 4 °C but could survive up to fourteen days or more in other tested media (chicken liver juice, beef liver juice, chicken juice and beef juice) at 4 °C [1]. However, *Campylobacter* strains can also survive for long periods in nutrient-poor environment despite losing cultivability [11]. Transcriptomic analysis of these bacteria during lower temperatures provides insight into their survival mechanisms and is a better approach for studying gene expression in variable environments [24]. Furthermore, changes in the incubation period, medium composition, temperature and atmospheric conditions alters expression in *Campylobacter* genomes [9,12,13]. In the current study, RNA-seq revealed that a large number of genes in two *Campylobacter* spp. were impacted by temperature fluctuations (from 42 °C to 4 °C). At 4 °C, medium composition (CJ vs. MHB) and incubation time also altered the *Campylobacter* transcriptome. 

In this study, relatively few orthologous genes were differentially expressed in both *Campylobacter* spp. when data were compared for temperature, medium formulation and incubation time. For example, our results show that *mreB*, which is involved in bacterial cell shape, was upregulated for *C. coli* but downregulated for *C. jejuni*. Temperature fluctuations are known to differentially impact *Campylobacter* gene expression; for example, the transcriptomes of *C. coli* and *C. lari* showed considerable variability in response to heat stress [15]. 

The oxidative stress response is reportedly a component of the *Campylobacter* response to cold shock [10,11,25], and the oxidative stress response genes *katA* and *sodB*, were previously upregulated at low temperatures in *C. jejuni* [25]. A previous study demonstrated that *trxB*, which encodes thioredoxin-disulfide reductase, had a potential role in the response of *C. jejuni* to oxidative stress [26]; however, in the current study, *trxB* was downregulated for both *C. jejuni* and *C. coli* in CJ and MHB media at all incubation times. Although *msrP* was downregulated in *C. jejuni* OD2-67 at all incubation times and in both media, it was either downregulated or unaltered in *C. coli* HC2-48. MsrP helps to repair oxidized proteins in the bacterial envelope during oxidative stress [27], and the oxidative stress response during cold shock might vary with the *Campylobacter* strain, medium and time of incubation. Although temperature fluctuations were closely monitored in this study, the exposure of bacterial samples to atmospheric oxygen during sample processing might cause in discrepancies in the oxidative stress response and gene expression. The iron concentration in media also influences the expression of genes involved in oxidative stress; for example, *katA, cj1386, ahpC* and *trxB* were repressed by iron [28]. Likewise, iron ions also reported to play role in the formation of oxygen radicals mediated by Fenton reaction in the bacterial cells [29,30,31,32,33]. Thus, variation in the iron content of CJ might have impacted *katA* expression in the current study. Interestingly, upregulation of *fur* (transcriptional repressor) was found in MHB for *C. coli* HC2-48 {Cc48_MHB_(0.5 h, 24 h, 48 h) vs. Cc48_MHB_0h} and in CJ for *C. jejuni* OD2-67 {Cj67_CJ_(0.5 h, 24 h, 48 h) vs. Cj67_CJ_0h} and might explain the reduced expression of genes related to oxidative stress and iron acquisition [28,34]. Meanwhile, *perR,* a transcriptional regulator that also plays role in oxidative stress and iron metabolism, was found upregulated in CJ for *C. coli* HC2-48 at 0.5 h {for both Cc48_CJ_0.5h/Cc48_42 and Cc48_CJ_0.5h/Cc48_MHB_0.5h) [28,34]. 

ClpB (AAA family ATPase) functions in various stress responses (oxidative, heat shock, starvation) and virulence in bacteria [2,35]. In our study, *clpB* was significantly downregulated in both *Campylobacter* spp. at low temperatures as compared to 42 °C in CJ and MHB. In a previous report, *clpB* expression in *C. jejuni* was induced by heat shock and repressed at low temperatures in MHB [13]. It was proposed that heat shock proteins such as ClpB, GroEL, GrpE, HrcA, CbpA, HspR, DnaK and GroES might not be required for survival at lower temperatures [13]. In the current study, these genes were generally downregulated or unaltered for the two *Campylobacter* strains. Hence, downregulation of heat shock response proteins might be common for *Campylobacter* spp. at temperatures lower than the optimum for growth. 

A number of flagellar genes (*flaG, fliS, flgG, flgP, flgN*) were downregulated in the two *Campylobacter* spp. in both CJ and MHB and at all incubation times. A few flagellar genes (e.g., *fliH, fliN* and *fliW*) were upregulated or unaltered in all experiments; interestingly, *fliK, fliW* and *fliY* were upregulated, but *flhB* and *flgC* were downregulated in CJ vs. MHB at one or more incubation times in *C. coli* HC2-48. For *C. jejuni* OD2-67, *flgM* was downregulated in CJ vs. MHB at 0 and 48 h. Downregulation of genes encoding flagellar proteins at suboptimal temperatures was previously reported for *C. jejuni* and may help the bacterium to conserve energy during adverse environmental conditions [13]. Another study reported upregulation of *flg* and downregulation of *fli* genes in *C. jejuni* at low temperatures [9]. In the current study, a greater number of flagellar genes was impacted in *C. coli* vs. *C. jejuni*; furthermore, variability in the expression of flagellar genes might be caused by variation in *Campylobacter* strains and medium formulations [9,11,13]. 

Suboptimal temperatures impacted the expression of *Campylobacter* genes that function in translation and ribosome structure [13]. In the current study, the expression of genes with roles in translation and ribosomal structure/biogenesis was highly represented in both *C. coli* and *C. jejuni*. A large proportion of translation/ribosome genes was upregulated in *C. coli* HC2-48 but downregulated in *C. jejuni* OD2-67 (Figure 3). Maintenance of cellular function during cold temperatures is essential and preserves the functionality of translational machinery [13], and the expression of these genes varies among *Campylobacter* spp. 

Genes for energy production and conservation were highly upregulated in both *Campylobacter* strains in this study; however, genes with significant expression in both strains at all incubation times and all medium formulations were limited. For example, *ppa* and *petC* encoding inorganic diphosphatase and cytochrome C1, respectively, were common upregulated genes for *C. coli* HC2-48 at 4 °C at all incubation times; however, *ppa* was unaltered, and *petC* was generally downregulated in *C. jejuni* OD2-67. Other genes including *atpB, ccpA* (cytochrome-c peroxidase), *hdrC* (FAD-binding oxidoreductase), *lldP* (L-lactate permease), *nuoA* (NADH quinone oxidoreductase, subunit 3) and *nuoM* (NADH quinone oxidoreductase, subunit M) were significantly upregulated in *C. jejuni* OD2-67 and were generally unaltered or significantly upregulated in *C. coli* HC2-48. These discrepancies show the interspecific variation in the transcriptome of *Campylobacter* spp. with respect to genes involved in energy production and conversion. In a prior report, the upregulation of *lldP* and other genes involved in macromolecule transport in *C. jejuni* was proposed to help the bacterium to acquire cryoprotectants that enhance survival at low temperatures [13]. In this study, DEGs related to amino acid transport and metabolism were less frequent and mostly downregulated. In contrast, genes involved in cell wall/membrane/envelope biogenesis were generally upregulated in the current study, which supports previous reports indicating that modifications in cell membrane structure might function in the cold-shock response [9,11,13]. 

Multiple genes encoding the type VI secretion system (T6SS) were upregulated in *C. jejuni* OD2-67 at 4 °C in CJ and MHB at all incubation times. A previous study reported that the T6SS enhanced the oxidative stress response, host colonization and virulence of *Campylobacter* strains [36,37]. Among the genes related to twin arginine translocation (TAT) system and Sec dependent pathways, *tatC* was generally upregulated in *C. coli* HC2-48, whereas *secE* and *secF* were upregulated or unaltered, and *secG* was downregulated or unaltered. Hence, it seems probable that translocation/secretion systems are important in *C. coli* HC2-48 at 4 °C. Meanwhile, *secY* was upregulated and *secE* was downregulated at one or more time points in *C. jejuni* OD2-67. No significant change in expression was observed for *tat* genes in *C. jejuni* OD2-67. 

In this study, many *Campylobacter* genes were differentially expressed in the two medium formulations at 4 °C. However, a previous report using microarrays identified only eight genes with significantly different expression in CJ as compared to brain heart infusion (BHI) medium at 5 °C [12]. This discrepancy might be attributed to the higher sensitivity of RNA-seq as compared to microarray analysis [28]. Despite the high number of DEGs identified in the present study, we did not identify a common gene in *C. coli* HC2-48 that was differentially expressed in CJ vs. MHB medium at all incubation times. Only one gene *A0W68_RS09275* (EexN family lipoprotein) was significantly expressed (downregulated) at all incubation times for *C. jejuni* OD2-67 and was plasmid-encoded. Hence, medium composition is unlikely a defining factor in differential expression of *Campylobacter* genes at low temperatures for prolonged time periods. In a previous report, *luxS* was upregulated in CJ vs. BHI and was proposed to function in adaptation to the CJ environment [12]. In the current study, *luxS* had a positive-fold-change value for all incubation times in *C. coli* HC2-48 but was only significantly upregulated at 48 h; in contrast, *luxS* expression was not differentially regulated in *C. jejuni* OD2-67. A gene related to iron transport (*A0W68_RS08550*, FTR1 family iron permease) was significantly upregulated for all incubation times except 48 h in *C. jejuni* OD2-67 cultivated in CJ. At one or more incubation times, a gene *torD* (cytoplasmic chaperone TorD) was differentially regulated in CJ vs. MHB in both *Campylobacter* strains; it was upregulated at 4 °C when compared to 42 °C in *C. coli* HC2-48 but downregulated for *C. jejuni* OD2-67 in both medium formulations. TorD reportedly functions in maturation of prokaryotic molybdoenzyme TorA [38], which is involved in electron transfer during anaerobic respiration [39]. 

A previous study identified various essential genes in *C. jejuni* for survival at low temperatures in nutrient-rich and nutrient-poor media [10]. Among the identified genes, *trxC* was described as having a fundamental role in the oxidative stress response [10]. In the current study, *trxC* was generally downregulated at 4 °C as compared to 42 °C in the two *Campylobacter* spp. and in both CJ and MHB. Two-component signal transduction systems have also been described as essential for survival at low temperatures [10]. In the two *Campylobacter* strains used in this study, the DccRS two-component system was notable, and *dccS* was significantly upregulated at most incubation times and in both media at 4 °C. Other upregulated genes in one or both *Campylobacter* strains included *czcD* (cation transporter), *hisC* (histidinol-phosphate transaminase) and *purN* (phosphoribosylglycinamide formyltransferase). 

Differential expression of various genes happened immediately after the temperature shifted from 42 °C to 4 °C; furthermore, some genes continued to be expressed at low temperatures, potentially due to differences in medium composition. In CJ, the number of significantly expressed genes decreased as time of incubation increased for *C. coli* HC2-48. Similarly, fewer genes were differentially expressed at 48 h (Cj67_CJ_48h/Cj67_CJ_0h) in *C. jejuni* OD2-67. However, unlike samples in CJ, a rapid decline in DEGs was not observed in MHB. No distinctive trends were observed for differences in expression with incubation time in the two media; for example, genes involved in motility and translation (ribosomal proteins) that were differentially expressed at 4 °C vs. 42 °C were not differentially expressed at other incubation times when compared to 0 h. Consequently, the expression of flagellar genes might not revert to original levels as suggested previously [13]. 

Many of the DEGs related to energy production/conversion in *C. coli* HC2-48 in MHB were upregulated after 0 h, which supports the contention that these functions are critical to survival at low temperatures [9,13]. Upregulation of *maf* and downregulation of *ftsW* indicate potential disruption of cell division since Maf inhibits septum formation and FtsW helps to recruit the transpeptidase FtsI to the division site [40,41]. Downregulation of *AR446_RS01805* (RNA pyrophosphoryl hydrolase) with respect to incubation time indicates that lower temperatures might alter degradosome activity and increase mRNA stability. RNA pyrophosphoryl hydrolase functions in mRNA degradation [42], and inactivation of the degradosome during stress has been reported [43]. 

In summary, RNA-seq revealed that a large number of genes in two *Campylobacter* spp. were impacted by temperature (4 °C vs. 42 °C). Genes related to cellular motility and the ribosome were impacted by 4 °C in *C. coli* HC2-48 and *C. jejuni* OD2-67, respectively. Variations in gene expression were observed as a function of incubation time, but a complete reversal of gene expression was not observed during prolonged incubation (48 h) at 4 °C. Although multiple genes were significantly expressed in CJ when compared to expression in MHB, no gene for *C. coli* HC2-48 and only one gene for C. *jejuni* OD2-67 was found significantly expressed at all incubation times. Hence, food matrix composition is not the sole determining factor for differential gene expression in *Campylobacter* spp. at low temperature. 

## Figures and Tables

**Table 1:** Overview of the *C. coli* HC2-48 (Cc48) and *C. jejuni* OD2-67 (Cj67) transcriptomes at 4 °C {(Cc48 or Cj67)_(MHB or CJ)_(0h or 0.5 h or 24 h or 48 h)} vs. 42 °C (Cc48_42 or Cj67_42). Genes showing ≥1.5 or ≤−1.5 fold-change and FDR < 0.05 were considered significant. *Campylobacter* strains were incubated in MHB and CJ.

Medium	MHB				CJ			
Time of Incubation at 4 °C	0 h	0.5 h	24 h	48 h	0 h	0.5 h	24 h	48 h
*C. coli* HC2-48, Total genes analyzed = 1651								
Number of regulated genes	350	348	323	325	233	307	183	309
Upregulated (%)	49.14%	52.01%	45.82%	54.46%	49.79%	51.47%	53.55%	50.81%
Downregulated (%)	50.85%	47.99%	54.18%	45.54%	50.21%	48.53%	46.45%	49.19%
*C. jejuni* OD2-67, Total genes analyzed = 1774								
Number of regulated genes	317	353	374	332	270	356	377	309
Upregulated (%)	56.15%	47.03%	51.87%	50.90%	56.67%	57.02%	50.93%	57.93%
Downregulated (%)	43.85%	52.97%	48.13%	49.10%	43.33%	42.98%	49.07%	42.07%

**Table 2:** Common genes of *C. coli* HC2-48 showing significant changes in expression at all incubation times (0 h, 0.5 h, 24 h and 48 h) and in both MHB and CJ media at 4 °C as compared to the control (microaerobic conditions in MHB at 42 °C).

Functional Group	Upregulated Genes	Downregulated Genes
Energy production/conversion	*ppa*/inorganic diphosphatase *petC* (AR446_RS02380)/cytochrome c1	*prpC* (AR446_RS06780)/citrate synthase/methylcitrate synthase
Cell cycle control, cell division, chromosome partitioning	*mreB*/rod shape-determining protein	*pseA*/pseudaminic acid biosynthesis protein PseA
Amino acid transport/metabolism		*hisH*/imidazole glycerol phosphate synthase subunit HisH *dapF* (AR446_RS00670)/diaminopimelate epimerase *cysK* cysteine synthase A *pseC*/UDP-4-amino-4, 6-dideoxy-N-acetyl-beta-L-altrosamine transaminase *ycaD* (AR446_RS04050)/MFS transporter *glnH* (AR446_RS04215)/transporter-substrate-binding-domain-containing protein
Nucleotide transport/metabolism	*purl*/phosphoribosylformylglycinamidine synthase subunit PurL	*dut* (AR446_RS01050)/dUTP diphosphatase
Carbohydrate transport/metabolism		*pseB*/UDP-N-acetylglucosamine 4,6-dehydratase (inverting) ^#^
Coenzyme transport/metabolism		*hemE* (AR446_RS02015)/uroporphyrinogen decarboxylase
Lipid transport/metabolism		*cdsA* (AR446_RS01495)/phosphatidate cytidylyltransferase *prpE* (AR446_RS06790)/propionyl-CoA synthetase *dxr*/1-deoxy-D-xylulose-5-phosphate reductoisomerase *acs* acetate—CoA ligase
Translation, ribosomal structure/ biogenesis	*rplM*/50S ribosomal protein L13 *rplQ*/50S ribosomal protein L17 *rpsI*/30S ribosomal protein S9 *hisS* (AR446_RS04490)/histidine--tRNA ligase *mnmE* tRNA uridine-5 carboxymethylaminomethyl(34) synthesis GTPase MnmE	*tet(O)* tetracycline resistance ribosomal protection protein Tet(O)
Transcription	*rpoA* (AR446_RS00265)/DNA-directed RNA polymerase subunit alpha	
Cell wall/membrane/envelope biogenesis	*rlpA*/septal ring lytic transglycosylase RlpA family protein	
Cell motility		*flhB*/flagellar biosynthesis protein FlhB *fliD*/flagellar filament capping protein FliD *flgL*/flagellar hook-associated protein FlgL *flgG*/flagellar basal-body rod protein FlgG *flaG* (AR446_RS05375)/flagellar protein FlaG *fliS*/flagellar export chaperone FliS *flgH*/flagellar basal body L-ring protein FlgH *flgB*/flagellar basal body rod protein FlgB *flgF* (AR446_RS04770)/flagellar hook-basal body protein *flgK*/flagellar hook-associated protein FlgK *flgM* (AR446_RS00985) */flagellar biosynthesis anti-sigma factor FlgM *flgN* (AR446_RS00980) */flagellar protein FlgN
Secondary metabolites biosynthesis/transport/catabolism		paaI (AR446_RS03420)/PaaI family thioesterase
Function unknown	AR446_RS07500/hypothetical protein *lptF* (AR446_RS04170)/LptF/LptG family permease	*cj1450* (AR446_RS01055)/hypothetical protein *actP* (AR446_RS08050)/cation acetate symporter AR446_RS00045/hypothetical protein AR446_RS07515/hypothetical protein AR446_RS08285/hypothetical protein AR446_RS01490/hypothetical protein rny/ribonuclease Y

* Annotated within ‘function unknown’ category in eggNOG vs. 5.0. ^#^ Also annotated in cell wall/membrane/envelope biogenesis.

**Table 3:** DEGs in *C. jejuni* OD2-67 that were significantly expressed in MHB and CJ at 4 °C and at all incubation times (0 h, 0.5 h, 24 h and 48 h) as compared to the control (microaerobic conditions in MHB at 42 °C).

Functional Group	Upregulated Genes	Downregulated Genes
Energy production/ conversion	*nuoA*/NAD(P)H-quinone oxidoreductase subunit 3 lldP/L-lactate permease *nuoM*/NADH-quinone oxidoreductase subunit M *atpB*/F0F1 ATP synthase subunit A *ccpA*/cytochrome-c peroxidase *hdrC*/FAD-binding oxidoreductase	*aldA*/aldehyde dehydrogenase *msrP*/protein-methionine-sulfoxide reductase catalytic subunit MsrP
Amino acid transport/ metabolism	aroQ/type II 3-dehydroquinate dehydratase	*map*/type I methionyl aminopeptidase *dapA*/4-hydroxy-tetrahydrodipicolinate synthase *leuA*/2-isopropylmalate synthase *trpE* * (A0W68_RS01665)/anthranilate synthase component I family protein
Coenzyme transport/metabolism	*Pcm*/protein-L-isoaspartate (D-aspartate)O-methyltransferase	
Translation, ribosomal structure/biogenesis	*ridA* (A0W68_RS03730)/RidA family protein	*rim*/16S rRNA processing protein RimM *rpsM*/30S ribosomal protein S13 *rpsK*/30S ribosomal protein S11
Transcription	A0W68_RS06530/response regulator transcription factor	
Replication, recombination, repair	*dnaN* (A0W68_RS00010)/DNA polymerase III subunit beta *hup* (A0W68_RS04770)/HU family DNA-binding protein	
Cell wall/membrane/envelope biogenesis	*pseF*/pseudaminic acid cytidylyltransferase A0W68_RS0595/hypothetical protein *lspA* (A0W68_RS01740)/lipoprotein signal peptidase	*cfa* (A0W68_RS06140)/class I SAM-dependent methyltransferase
Cell motility	*flip*/flagellar type III secretion system pore protein FliP methyl accepting chemotaxis proteins (A0W68_RS08070, A0W68_RS00090)	*fliS*/flagellar export chaperone FliS *flgN* */flagellar protein FlgN
Post-translational modification, protein turnover and chaperones	*clpP*/ATP-dependent Clp endopeptidase proteolytic subunit ClpP	*grpE*/nucleotide exchange factor GrpE *cbpA* (A0W68_RS06375)/DnaJ family protein
Inorganic ion transport and metabolism	Sodium dependent transporter (A0W68_RS04880, A0W68_RS02830) *ctf*/non-heme ferritin	*futA1* (A0W68_RS00880)/Fe(3+) ABC transporter substrate-binding protein
Secondary metabolites biosynthesis/transport/catabolism	*fahA* (A0W68_RS00100)/fumarylacetoacetate hydrolase family protein	
Function unknown	A0W68_RS04155/hypothetical protein *arsP* /organoarsenical efflux permease ArsP *pseE1* (A0W68_RS07185)/motility associated factor glycosyltransferase family protein *yccA* (A0W68_RS01145)/Bax inhibitor-1/YccA family protein A0W68_RS01955/SPOR-domain-containing protein A0W68_RS02215/DUF374-domain-containing protein A0W68_RS07500/hypothetical protein	A0W68_RS01890/hypothetical protein A0W68_RS02435/YkgJ family cysteine cluster protein *ciaC*/invasion antigen CiaC *uup* (A0W68_RS04650)/ABC-F family ATP-binding-cassette-domain-containing protein A0W68_RS04080/cupin-domain-containing protein A0W68_RS07830/LysR family transcriptional regulator *dba*/disulfide bond formation protein Dba A0W68_RS00330/membrane protein), A0W68_RS06720/hypothetical protein
Intracellular trafficking, secretion and vesicular transport	*dctA* (A0W68_RS06185)/cation:dicarboxylase symporter family transporter	*yajC*/preprotein translocase subunit YajC
Defense mechanism		*macB* (A0W68_RS02860)/ABC transporter permease *yokD* (A0W68_RS06985)/aminoglycoside N(3)-acetyltransferase

* Annotated within ‘function unknown’ category in eggNOG vs. 5.0.

**Table 4:** Genes having fold-change values with FDR < 0.05 in both *C. coli* HC2-48 and *C. jejuni* OD2-67 at all incubation times (0 h, 0.5 h, 24 h and 48 h) in MHB and CJ at 4 °C as compared to the control (microaerobic condition in MHB at 42 °C). Only *fliS* and *flgN* had significant expression (fold-change values ≥ 1.5 or ≤ −1.5 and FDR < 0.05) in both strains at all sampling times in both media.

Functional Group	Upregulated Genes	Downregulated Genes
Energy production/conversion		*trxB*/thioredoxin-disulfide reductase
Cell cycle control, cell division, chromosome partitioning	*mreB* ^#^/rod shape-determining protein	
Coenzyme transport/ metabolism		*hemE*/uroporphyrinogen decarboxylase
Translation, ribosomal structure/biogenesis	*rplM*/50S ribosomal protein L13	
Cell motility		*flaG*/flagellar protein FlaG *fliS*/flagellar export chaperone FliS *flgG*/flagellar basal-body rod protein FlgG *flgP* */flagellar assembly lipoprotein FlgP *flgN* */flagellar protein FlgN
Post translational modification, chaperones		*clpB*/AAA family ATPase
Function unknown		AR446_RS06165/A0W68_RS01890/hypothetical protein

* Annotated as Function Unknown by eggNOG 5.0. ^#^ Upregulated for *C. coli* HC2-48 but downregulated for *C. jejuni* OD2-67.

## Data Availability

All the original sequence reads for this experiment accessible in the Sequence Read Archive (SRA) database within Bio project id: PRJNA828109 (https://www.ncbi.nlm.nih.gov/sra?linkname=bioproject_sra_all&from_uid=828109, accessed on 9 March 2020).

## Supplementary Materials

The following supporting information can be downloaded at: https://www.mdpi.com/article/10.3390/pathogens12070960/s1.
